# Modulation of the mitochondrial Ca^2+^ uniporter complex subunit expression by different shear stress patterns in vascular endothelial cells

**DOI:** 10.14814/phy2.15588

**Published:** 2023-02-08

**Authors:** Akshar Patel, Julia G. Pietromicca, Manigandan Venkatesan, Soumya Maity, Jonathan E. Bard, Muniswamy Madesh, B. Rita Alevriadou

**Affiliations:** ^1^ Vascular Mechanobiology Laboratory, Department of Biomedical Engineering, and Center for Cell, Gene, and Tissue Engineering University at Buffalo – The State University of New York Buffalo New York USA; ^2^ Department of Medicine, Center for Mitochondrial Medicine University of Texas Health San Antonio San Antonio Texas USA; ^3^ Genomics and Bioinformatics Core, Jacobs School of Medicine and Biomedical Sciences University at Buffalo – The State University of New York Buffalo New York USA

**Keywords:** calcium signaling, endothelial dysfunction, hemodynamics, mitochondrial dynamics, oxidative stress

## Abstract

Mitochondrial calcium (_m_Ca^2+^) uptake occurs via the Mitochondrial Ca^2+^ Uniporter (MCU) complex and plays a critical role in mitochondrial dynamics, mitophagy, and apoptosis. MCU complex activity is in part modulated by the expression of its regulatory subunits. Cardiovascular disease models demonstrated altered gene/protein expression of one or multiple subunits in different cells, including vascular endothelial cells (ECs). MCU complex activity was found necessary for stable flow (s‐flow)‐induced mitophagy and promotion of an atheroprotective EC phenotype. Disturbed flow (d‐flow) is known to lead to an atheroprone phenotype. Despite the role of MCU in flow‐regulated EC function, flow‐induced alterations in MCU complex subunit expression are currently unknown. We exposed cultured human ECs to atheroprotective (steady shear stress, SS) or atheroprone flow (oscillatory shear stress, OS) and measured mRNA and protein levels of the MCU complex members. SS and OS differentially modulated subunit expression at gene/protein levels. Protein expression changes of the core MCU, _m_Ca^2+^ uptake 1 (MICU1) and MCU regulator 1 (MCUR1) subunits in SS‐ and OS‐exposed, compared to static, ECs suggested an enhanced _m_Ca^2+^ influx under each flow and a potential contribution to EC dysfunction under OS. In silico analysis of a single‐cell RNA‐sequencing dataset was employed to extract transcript values of MCU subunits in mouse carotid ECs from regions exposed to s‐flow or d‐flow. *Mcu* and *Mcur1* genes showed significant differences in expression after prolonged exposure to each flow. The differential expression of MCU complex subunits indicated a tight regulation of the complex activity under physiological and pathological hemodynamic conditions.

## Graphical abstract

Patel et al. demonstrated that flow patterns differentially modulate the Mitochondrial Ca^2+^ Uniporter (MCU) subunit expression at the gene and protein levels. Protein expression changes of the core MCU, mitochondrial Ca^2+^ uptake 1 (MICU1) and MCU regulator 1 (MCUR1) subunits in atheroprotective flow‐exposed and atheroprone flow‐exposed, compared to static, ECs suggested an enhanced mitochondrial Ca^2+^ influx under each flow and a potential contribution to EC dysfunction under atheroprone flow. For in vivo evidence of mechanoregulation of the MCU subunit gene expression, in silico analysis of a single‐cell RNA‐sequencing dataset was employed to extract transcript values of MCU subunits in mouse carotid ECs from regions exposed to atheroprotective versus atheroprone flow.

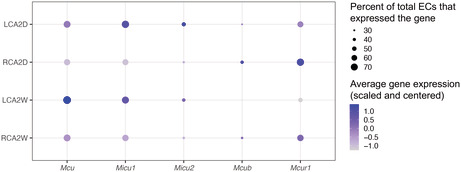

## 1 INTRODUCTION

Mitochondrial Ca^2+^ (_m_Ca^2+^) uptake is an electrogenic process driven by the negative membrane potential (ΔΨ_m_) across the inner mitochondrial membrane and occurs via the Mitochondrial Ca^2+^ Uniporter (MCU), a Ca^2+^‐selective ion channel (Baughman et al., 2011; De Stefani et al., 2011; Kirichok et al., 2004; Murgia & Rizzuto, 2015). By controlling the Ca^2+^ influx rate to the mitochondria and, hence, the _m_Ca^2+^ concentration ([Ca^2+^]_m_), the MCU regulates mitochondrial respiration, ATP production, mitochondrial dynamics and mitophagy, and cell survival (Bertero & Maack, 2018; De Stefani et al., 2016; Duchen, 1999; Hajnoczky et al., 2006; Murgia & Rizzuto, 2015; Pathak & Trebak, 2018). The MCU is a heteromeric protein complex that consists of a Ca^2+^‐conducting core protein, also called MCU, and regulatory proteins. The regulatory proteins include the _m_Ca^2+^ uptake (MICU) family (MICU1‐3), MCU dominant‐negative β‐subunit (MCUb), MCU regulator 1 (MCUR1), essential MCU regulator (EMRE), and solute carrier 25A23 (SLC25A23). The structure and functional role of each member of the MCU complex were recently reviewed (Alevriadou et al., 2021; Garbincius & Elrod, 2022; Pallafacchina et al., 2021). Briefly, the complex consists of 4 MCU and 4 EMRE subunits, and each EMRE interacts with an MCU and binds to MICU1. MICU1/2 form heterodimers that extend their Ca^2+^‐binding domains into the intermembrane space (IMS) and, by sensing changes in the local IMS Ca^2+^ concentration (same as the local cytosolic Ca^2+^ concentration, [Ca^2+^]_c_), they control the MCU complex activity. MCUb is a dominant‐negative form of MCU that interacts with MCU forming a heterooligomer. MCUR1 binds to MCU and EMRE and acts as a scaffold factor for MCU channel function. Besides its regulation by [Ca^2+^]_c_, the MCU complex is also regulated by transcriptional, posttranscriptional, and posttranslational modifications of its subunits (Alevriadou et al., 2021; Garbincius & Elrod, 2022; Nemani et al., 2018). Both in vivo and in vitro models of disease, including cardiovascular disease (CVD), demonstrated alterations in mRNA and/or protein levels of specific MCU complex subunits in different cell types, compared to controls, which were found to contribute to cell dysfunction and disease development (Chen et al., 2017, 2021; Natarajan et al., 2020; Wright et al., 2017; Zaglia et al., 2017; Zhang et al., 2021).

Atherosclerosis, the underlying cause of CVD, occurs in a site‐specific manner at outer walls of arterial bifurcations and inner walls of curvatures, where blood flow is disturbed (d‐flow; Asakura & Karino, 1990; Brown et al., 2016; Glagov et al., 1988; Malek et al., 1999). D‐flow is typically simulated in vitro as oscillatory flow (corresponding to oscillatory shear stress, OS; Rezvan et al., 2011). In contrast, flow in straight arterial segments is undisturbed, stable flow (s‐flow; Asakura & Karino, 1990; Brown et al., 2016; Glagov et al., 1988; Malek et al., 1999). S‐flow is pulsatile, but is commonly simulated in vitro as steady flow corresponding to steady shear stress, SS, at a value equal to the time average of pulsatile shear stress (Rezvan et al., 2011). Investigators have shown in vivo (and in vitro) that d‐flow (OS) is pro‐atherogenic by promoting an oxidative and inflammatory state in vascular endothelial cells (ECs), whereas s‐flow (SS) is anti‐atherogenic by inhibiting EC oxidative stress and inflammation (Davies et al., 2013; Gimbrone Jr. & Garcia‐Cardena, 2016; Nigro et al., 2011). The atheroprotective effect of s‐flow is majorly due to upregulation of the transcription factor Krüppel‐like factor 2 (*Klf2*) and its downstream target endothelial nitric oxide (NO) synthase (*eNOS*) that produces the vasodilator NO (Wang et al., 2006, 2010). It was recently shown in vivo (and in vitro) that the MCU‐mediated _m_Ca^2+^ influx is necessary for s‐flow (SS)‐induced EC mitophagy, which contributes to *Klf2* expression and the resultant suppression of inflammation (Coon et al., 2022). In contrast, ECs exposed to d‐flow (OS), due to their defective mitophagy, did not upregulate *Klf2* and instead expressed inflammatory markers (Coon et al., 2022).

Our group recently showed that short‐term (mins) exposure of cultured human ECs transduced to overexpress MCU (ECs with increased MCU complex activity/_m_Ca^2+^ uptake) to SS elicited a [Ca^2+^]_m_ response that included oscillations (Patel et al., 2022). [Ca^2+^]_m_ oscillations were absent in either MCU‐transduced ECs that were kept static or SS‐exposed βGal‐transduced (or untransduced) ECs suggesting that altered MCU complex activity in combination with flow has a major impact on flow‐induced [Ca^2+^]_m_ signaling. The frequency of [Ca^2+^]_m_ oscillations in SS‐exposed MCU‐transduced ECs depended on mitochondrial reactive oxygen species (mROS) flashes and ΔΨ_m_ flickers, as well as on Piezo1 and eNOS activities (Patel et al., 2022). In an earlier study, MCU complex activity was found critical in maintaining the normal cytosolic Ca^2+^ signaling in ECs exposed to SS (Scheitlin et al., 2016).

Since the MCU complex activity is critical in flow‐regulated EC Ca^2+^ signaling and function, it is important to assess how different flow patterns affect the MCU complex subunit expression levels, which, at least in part, regulate complex activity. In this study, we hypothesized that prolonged SS and OS may differentially modulate the gene/protein expression of MCU complex members in cultured human ECs. Our data showed significant differences in the expression of *MCU*, *MICU1*, *MCUb* and *MCUR1* genes and MCU, MICU1 and MCUR1 proteins among ECs exposed to 24 h of static, SS or OS. Based on the functional role of MCU complex members, the observed changes in protein expression suggested an enhanced _m_Ca^2+^ influx in ECs exposed to each flow compared to static and a potential causative link to EC dysfunction under OS. To compare our in vitro findings with in vivo evidence, a published single‐cell RNA‐sequencing (scRNA‐seq) dataset (Andueza et al., 2020) was reanalyzed and transcript levels of MCU complex subunits from mouse carotid ECs exposed to s‐flow versus d‐flow were extracted. The in vivo evidence showed significant differences in *Mcu* and *Mcur1* expression between the two flows. A disagreement between in vivo and in vitro gene expression profiles can be attributed to factors such as differences in cell origin, duration of treatment, and fluid forces. This study points to the need for a better understanding of the mechanosignaling that regulates the MCU complex activity, an important player in EC dysfunction and CVD.

## 2 MATERIALS AND METHODS

### 2.1 EC culture and exposure to SS or OS

Pooled primary HUVECs (C2519AS; Lonza) were grown in complete EC growth medium (EGM™ Endothelial cell growth medium bulletkit™: CC‐3124; Lonza) in a tissue culture incubator at 37°C in an atmosphere of 5% CO_2_/95% air. Plastic parallel‐plate flow chamber slides with high optical quality (μSlide 0.6 luer, 1.5 coverslip: 80186; ibidi) were coated with 3% fibronectin solution for 40 min. ECs of passage 3–5 were seeded on the fibronectin‐coated chamber slides at a seeding density of 80,000–100,000 cells/cm^2^. After 24 h, EC monolayers had reached 90% confluency. ECs were preincubated with media consisting of EBM (EBM™ Endothelial cell growth basal medium, phenol red‐free: CC‐3129; Lonza) supplemented with 2% FBS (fetal bovine serum: CC‐4133; Lonza) and antibiotics (CC‐4083; Lonza) for 4 h for cells to become quiescent and equilibrate with the perfusion media. EC monolayers were then attached to an ibidi pump system in a serially‐connected manner with two flow chamber slides per fluidic unit to provide a protein sample adequate for analysis from each flow experiment. A master flow unit equipped with ibidi's “yellow‐green” perfusion set (length 50 cm, ID 6 mm and 10 ml reservoirs) was used to achieve SS of 15 dynes/cm^2^ (35.66 ml/min, 81.9 mbar) and a servant flow unit equipped with ibidi's “blue” perfusion set (length 15 cm, ID 0.8 mm and 10 ml reservoirs) to achieve OS of ±4 dynes/cm^2^ (0 ± 10.43 ml/min, 81.9 mbar). EC monolayers were exposed to either static, SS or OS using perfusion media of the same composition as the equilibration media for 24 h. Cell viability was assessed at the end of shear exposure and was greater than 90%.

### 2.2 Western blotting against MCU complex subunits

HUVECs in the flow chambers were washed twice with cold phosphate‐buffered saline and lysed using RIPA buffer (ab156034; Abcam) and HaltTM protease and phosphatase inhibitor cocktail, EDTA‐free (78441; ThermoFisher Scientific). Lysates were homogenized by passing through a 25‐gauge needle and centrifuged at 14,000*g* for 10 min. Equal amounts of protein (20 μg/lane) were separated on 4%–12% Bis‐Tris polyacrylamide gels (WG1402BOX; ThermoFisher Scientific), transferred to a PVDF membrane (1B24001; ThermoFisher Scientific) using iBlot 2 PVDF regular stacks (IB21001; ThermoFisher Scientific), and probed with antibodies specific for MCU (D2Z3B, 1:5000; Cell Signaling), MICU1 (D4P8Q, 1:3000; Cell Signaling); MICU2 (ab101465, 1:2000; Abcam), MCUb (MBS3223833, 1:2000; MyBioSource), MCUR1 (13706, 1:3000; Cell Signaling), TOMM20 (42406S, 1:3000; Cell Signaling), and ACTB (SC47778, 1:10,000; Santa Cruz Biotechnology). Signals were visualized via chemiluminescence (SuperSignal West Pico PLUS Chemiluminescent Substrate: 34578; ThermoFisher Scientific) and quantified using ImageJ (https://imagej.nih.gov/ij/).

### 2.3 Reverse transcription‐quantitative polymerase chain reaction (RT‐qPCR) for MCU complex subunits

Total RNA was extracted from HUVECs at the end of treatment using the RNeasy mini kit (74104; Qiagen). Reverse Transcription was performed using the iScript cDNA Synthesis Kit (1708890; Bio‐Rad). Quantitative real‐time PCR was performed in duplicate using the iTaq Universal SYBR Green Supermix (1725120; Bio‐Rad) and the CFX Connect Real‐Time PCR Detection System (1855201; Bio‐Rad). The PCR program consisted of an initial step at 95°C for 30 s, followed by 40 cycles of denaturation at 95°C for 3 s, annealing/extension at 60°C for 20 s, followed by melting at a gradient from 65 to 95°C. Gene expression was normalized to the housekeeping genes *GAPDH* and *ACTB*. Normalized fold expression was calculated and plotted using the ∆∆Ct method. All primer sequences were verified for specificity using Primer‐BLAST: *MCU*: 5′‐TTGCTCAGGCAGAAATGGAC‐3′ (forward) and 5′‐AGGCAAACAGGTGTTCCTTCT‐3′ (reverse), *MICU1*: 5′‐ACTGTGATGGCAATGGCGAA‐3′ (forward) and 5′‐TAAAGCGAAGTCCCAGGCAG‐3′ (reverse), *MICU2*: 5′‐AGAGACCTTGGCGATAAAGGG‐3′ (forward) and 5′‐ATCCAGAATGGGGTTTAGTGAGG‐3′ (reverse), *MCUB*: 5′‐AGAAGCTCATTCGGAAGCCAA‐3′ (forward) and 5′‐CCAGGAGTACACCCACCAC‐3′ (reverse), *MICUR1*: 5′‐ATGCCTTAGTGTGCTTACTGGA‐3′ (forward) and 5′‐TTCACATTCGCAATCTGAGACAT‐3′ (reverse), *GAPDH*: 5′‐CCACAGTCCATGCCATCAC‐3′ (forward) and 5′‐CCACCACCCTGTTGCTGTA‐3′ (reverse), and *ACTB*: 5′‐CCAACCGCGAGAAGATGA‐3′ (forward) and 5′‐CCAGAGGCGTACAGGGATAG‐3′ (reverse).

### 2.4 Bioinformatics analysis of a scRNA‐seq dataset

In Andueza et al. (2020), C57BL/6 mice were partially ligated to induce d‐flow in the left carotid artery (LCA) using the contralateral right carotid artery (RCA) exposed to s‐flow as control. At 2 days (2D) and 2 weeks (2W) post‐PCL, lumens of 10 LCAs (LCA2D and LCA2W) and 10 RCAs (RCA2D and RCA2W) were digested with collagenase to collect single cells for scRNA‐seq analysis. In our study, raw scRNA‐seq data available under the National Center for Biotechnology Information Bioproject accession PRJNA646233 were downloaded and reprocessed using the 10X Genomics software utility Cellranger version 7.0. The resulting cell‐to‐gene matrix files were used as input into the R package Seurat v4.1 and sample‐to‐sample integration workflow was performed. Cells shown to have greater than 20% mitochondrial transcript load were filtered from the analysis. The uniform manifold approximation and projection (UMAP) reduction and Louvain clusters were annotated following the clusters described by Andueza et al. Specifically, clusters E1–E8 of mouse carotid ECs were identified based on their expression of *Pecam1*, *Icam2*, *Cdh5*, and *Tie1*. ECs in E1–E4 clusters had higher expression levels of the mechanosensitive and atheroprotective genes *Klf2* and *Klf4* than ECs in E5–E8 clusters. In contrast, ECs in E5–E8 clusters had higher expression levels of the atheroprone genes *Ctfg*, *Serpine1*, *Edn1*, and *Thsp1*. Importantly, the E1–E4 clusters mostly comprised of ECs exposed to s‐flow in the RCA, whereas the E5–E8 clusters mostly comprised of ECs exposed to d‐flow in the LCA (Andueza et al., 2020). In this study, individual LogNormalized gene expression profiles for *Mcu*, *Micu1*, *Micu2*, *Mcub*, and *Mcur1* were plotted using Seurat's FeaturePlots, Vlnplots, and DotPlot functions. For the DotPlots, the default settings of scaled expression data were used. Differential expression for each gene in ECs of LCA versus RCA at 2D and 2W was calculated using Seurat's FindAllMarkers function.

### 2.5 Statistical analysis

Protein expression data of MCU, MICU1, MICU2, MCUb, MCUR1, and TOMM20 (normalized to ACTB) were presented as mean ± SE from *n* = 6–9 independent experiments for each condition tested, static, OS, and SS. Gene expression data of *MCU*, *MICU1*, *MICU2*, *MCUb*, and *MCUR1* (normalized to *GAPDH* and *ACTB*) were presented as mean ± SE from *n* = 3 independent experiments for each condition tested. Statistical differences were determined using one‐way analysis of variance (ANOVA) followed by Tukey's multiple comparisons post hoc test with *p* ≤ 0.05 indicating statistical significance. Statistical analyses were performed using GraphPad Prism 9 on a PC. Regarding the scRNA‐seq data analysis, differential expression of *Mcu*, *Micu1*, *Micu2*, *Mcub*, and *Mcur1* was tested between LCA and RCA at 2D and 2W using the built‐in function “FindMarkers” and “FindAllMarkers” in the R package Seurat, which by default uses the Wilcoxon rank‐sum test, a non‐parametric alternative to the two‐sample *t*‐test, followed by multiple‐comparison Bonferroni correction. Adjusted *p* values ≤0.05 indicated statistical significance.

## 3 RESULTS

### 3.1 Exposure of cultured ECs to SS and OS differentially modulated the protein and/or gene expression of select MCU complex subunits

**FIGURE 1:**
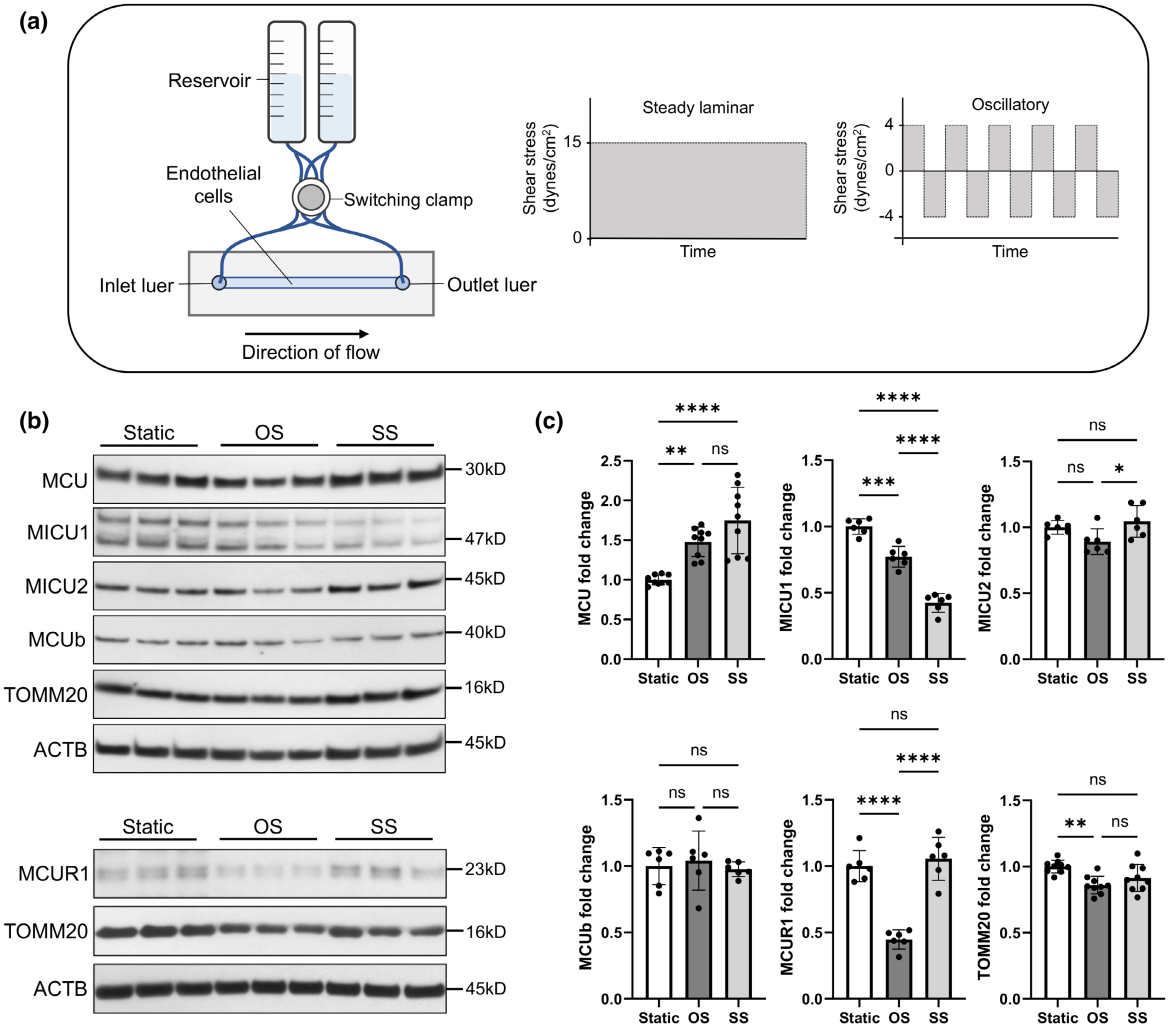
Using the ibidi pump system, it was found that SS and OS cause differential expression of MCU complex members in HUVECs. (a) Schematic diagram of the ibidi pump system to drive either steady laminar shear stress (SS) or oscillatory shear stress (OS) across ECs in a slide chamber. (b) Characteristic Western blots against the MCU complex proteins, MCU, MICU1, MICU2, MCUb, as well as the mitochondrial marker TOMM20 and loading control ACTB (20 μg of total protein/lane). (c) Expression levels were normalized to ACTB and plotted as mean ± SE of fold change from *n* = 6–9 independent experiments. ^ns^ *p* > 0.5; *p ≤ 0.5; **p ≤ 0.01; ***p ≤ 0.001; ****p ≤ 0.0001.

The ibidi pump system was used in this study to drive either steady unidirectional laminar flow (SS of 15 dynes/cm^2^) or oscillatory laminar flow (OS of ±4 dynes/cm^2^), as described under Section 2 (Figure 1a). Lysates of cultured HUVECs exposed to 24 h of either static (control) incubation, SS or OS from *n* = 6–9 independent experiments were analyzed for expression of MCU complex proteins, (core) MCU, MICU1, MICU2, MCUb, and MCUR1, as well as the mitochondrial marker TOMM20, and expression was normalized to that of ACTB (Figure 1b,c). Both flows caused a statistically significant upregulation of the core MCU expression and downregulation of the gatekeeper MICU1 expression compared to static. No significant difference in MCU was found between SS and OS. SS caused an additional downregulation of MICU1 compared to OS, but maintained the gatekeeper MICU2 at the static level. OS, but not SS, caused a significant downregulation of the MCU complex scaffolding protein MCUR1 compared to static. Expression of MCUb was not significantly different between the two flows. OS also caused a small, but significant, downregulation of TOMM20 compared to control (Figure 1c).

MICU1/2 play a gatekeeping role by preventing _m_Ca^2+^ uptake at low [Ca^2+^]_c_ and allowing efficient _m_Ca^2+^ uptake when [Ca^2+^]_c_ increases (Mallilankaraman et al., 2012b; Patron et al., 2014). The significant increase in core MCU expression and significant decrease in expression of the gatekeeper MICU1 in ECs exposed to either SS or OS suggest that either flow caused an increase in MCU complex activity/_m_Ca^2+^ uptake compared to static controls. This agrees with our recent work where SS applied on ECs was found to increase [Ca^2+^]_m_ levels above those in static ECs (Patel et al., 2022). Besides exhibiting an increase in MCU and a decrease in MICU1 expression relative to static, OS also caused a significant decrease in MCUR1 expression compared to both static and SS. A number of functions have been ascribed to MCUR1, but none has been studied in sheared ECs: MCUR1‐dependent MCU function is thought to be required for maintenance of bioenergetics and/or the mitochondrial permeability transition pore (mPTP; Chaudhuri et al., 2016; Mallilankaraman et al., 2012a; Paupe et al., 2015; Tomar et al., 2016). Overall, due to its ability to sense [Ca^2+^]_m_ and its physical interaction with the mPTP modulator cyclophilin D (CypD), MCUR1 may act as a switch to regulate other proteins (such as the cytochrome c oxidase and the mPTP) and cellular processes, which may or may not compete with its promotion of the MCU complex activity (Chaudhuri et al., 2016; Garbincius & Elrod, 2022).

**FIGURE 2:**
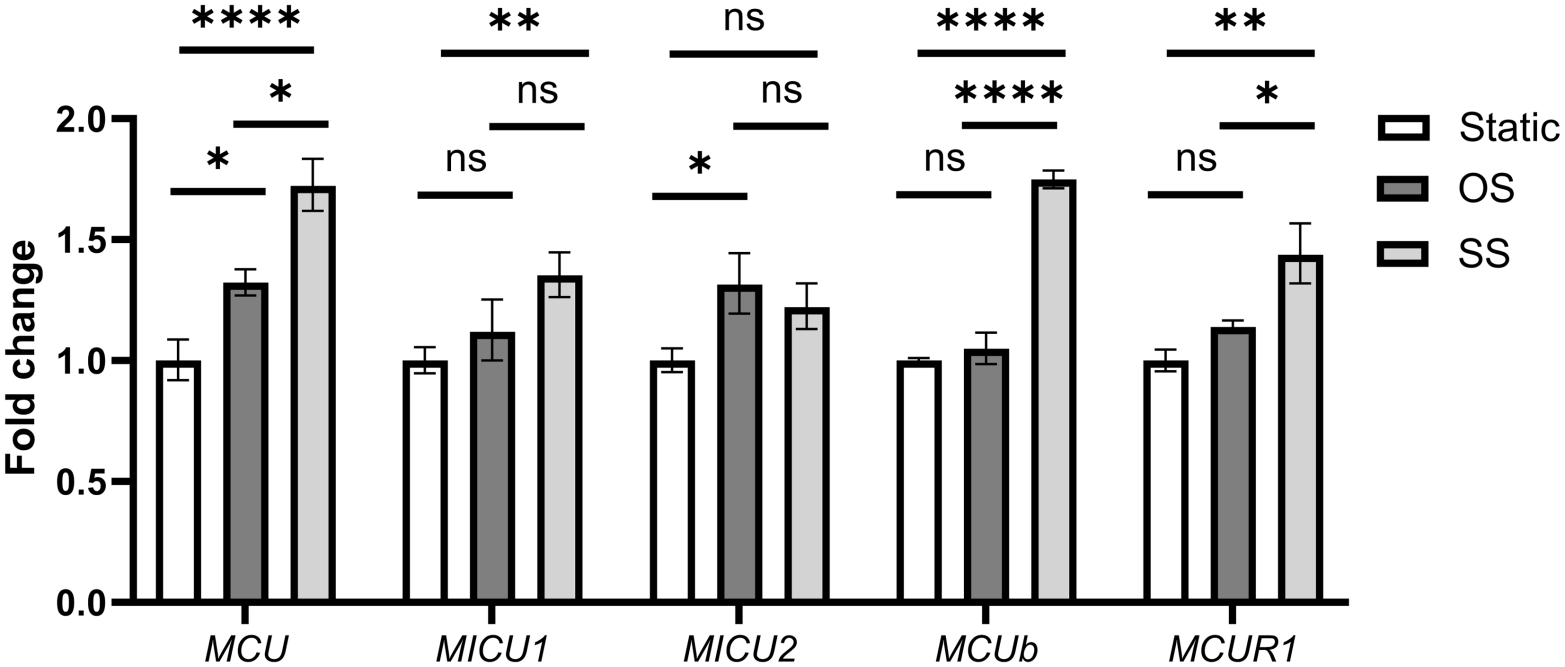
SS and OS caused differential expression of genes that encode MCU complex subunits in HUVECs. ECs were exposed to the flows using the ibidi pump system, total RNA was extracted, and RT‐qPCR was performed to quantify expression of genes *MCU*, *MICU1*, *MICU2*, *MCUb*, *MCUR1*, *GAPDH,* and *ACTB* (the latter two were used to normalize MCU complex subunit gene expression). Expression levels were plotted as mean ± SE of fold change from *n* = 3 independent experiments. ^ns^ *p* > 0.5; *p ≤ 0.5; **p ≤ 0.01; ***p ≤ 0.001; ****p ≤ 0.0001.

When ECs exposed to static, OS or SS were analyzed for MCU complex subunit gene expression, *MCU*, *MCUb* and *MCUR1* were found to differentially express between the two flows (Figure 2). The significant upregulation of *MCUb* and, possibly also of, *MCUR1* may be an attempt by the cell to counteract the significant increase in *MCU* expression in SS‐exposed, compared to static or OS‐exposed, ECs (Figure 2).

### 3.2 MCU complex subunit genes in ECs from a mouse PCL model were differentially affected by s‐flow (RCA) versus d‐flow (LCA)

**FIGURE 3:**
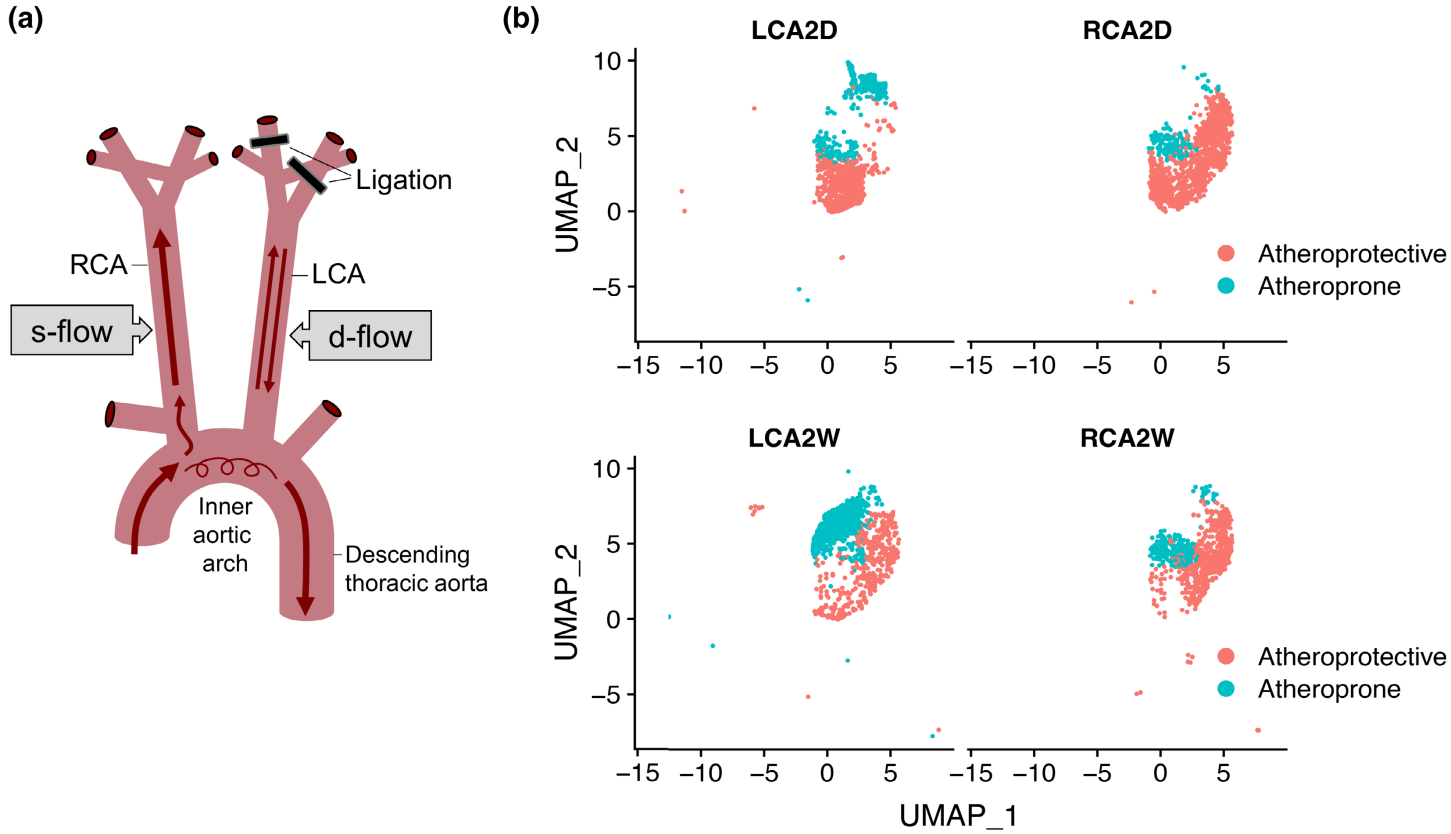
Analysis of scRNA‐seq data from a PCL mouse model showed that LCA and RCA contain a heterogeneous mixture of ECs, but the LCA contains mostly ECs with an atheroprone phenotype (the opposite is true for RCA). (a) Schematic diagram of the PCL model, in which three of the four branches of the LCA are ligated leading to development of d‐flow in LCA, whereas RCA maintains s‐flow. (b) EC clusters were grouped into “atheroprotective” versus “atheroprone”, based on relative expression of characteristic marker genes according to Andueza et al., and plotted by UMAP in LCA and RCA at 2 days (2D) and 2 weeks (2W) post‐PCL. Notice the enrichment of LCA in ECs with an atheroprone phenotype, especially at 2W.

Ligation of three of the four branches of the mouse LCA is known to induce d‐flow in the LCA, whereas flow in the RCA remains undisturbed s‐flow (Nam et al., 2009; Ni et al., 2010; Simmons et al., 2016; Figure 3a). In silico analysis of published (Andueza et al., 2020) scRNA‐seq data from a mouse PCL model was employed to detect transcript expression for each of the MCU complex subunits in ECs, as described under Section 2. Since EC clusters E1–E4 had higher expression levels of *Klf2* and *Klf4*, they were called “atheroprotective”, and the remaining E5–E8 clusters were called “atheroprone”. When atheroprotective and atheroprone clusters were plotted by UMAP in LCA and RCA at 2D and 2W, it demonstrated that each carotid artery contained a mixture of ECs with the two phenotypes (Figure 3b). However, the LCA mostly contained ECs with the atheroprone phenotype and the RCA mostly ECs with the atheroprotective phenotype; that trend became more pronounced at 2W compared to 2D post‐PCL confirming that d‐flow is a pro‐atherogenic stimulus (Figure 3b).

**FIGURE 4:**
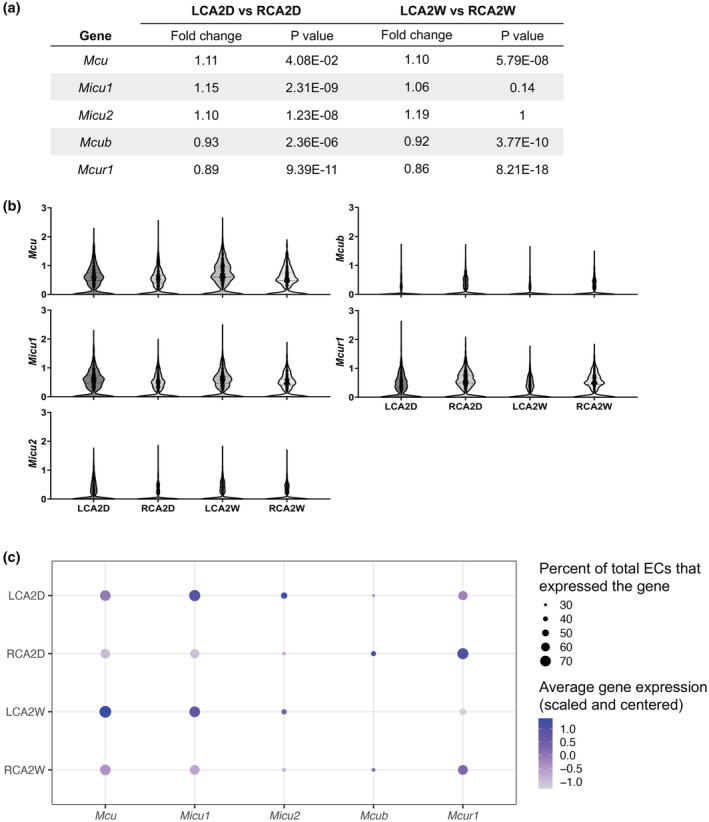
D‐flow and s‐flow exerted on ECs in the LCA and RCA, respectively, caused differential expression of genes that encode MCU complex subunits. (a) DEG values for *Mcu*, *Micu1*, *Micu2*, *Micub*, and *Mcur1* are listed as fold change in LCA versus RCA at 2 days (2D) and 2 weeks (2W) post‐PCL together with the corresponding adjusted *p* values. (b) Violin plots for each MCU complex subunit gene. (c) Dot plot shows the percent of ECs that expressed each gene and the scaled and centered expression level of that gene averaged across all ECs in the LCA and RCA at 2D and 2W. Briefly, the counts for each gene in each cell were first normalized and scaled using the R package Seurat's default LogNormalization and ScaleData functions. These functions implement a global‐scaling normalization to control for the variable depth of sequencing per cell, and then scale and center each gene measurement to have a mean of 0 and SD of 1. This value was reported as average gene expression (scaled and centered). Notice that the most noteworthy flow‐induced transcriptional changes were the upregulation of *Mcu* and downregulation of *Mcur1* by d‐flow (LCA) compared to s‐flow (RCA) at 2W post‐PCL.

Following the generation of differentially expressed gene (DEG) tables in Seurat and calculation of corrected *p* values, it was found that the *Mcu* expression was significantly higher and those of *Mcub* and *Mcur1* were significantly lower in LCA versus RCA at 2W (Figure 4a). All fold changes in LCA in 2D and 2W were relatively modest, within ±0.15 of RCA (Figure 4a). Violin plots for each gene demonstrated the relative differences between LCA and RCA at 2D and 2W (Figure 4b). To better visualize these differences, a dot plot of the percent of ECs that expressed a particular gene and the scaled and centered expression level of that gene averaged across all ECs in LCA and RCA at 2D and 2W was produced (Figure 4c). Approximately ≥70% of ECs had *Mcu* and *Micu1* transcripts; only *Mcu* had a significantly higher expression in LCA versus RCA at 2W (Figure 4a,c). *Mcur1* was expressed by a relatively high percentage of ECs, but its expression was significantly lower in LCA versus RCA at 2D and 2W. A relatively small percentage of cells (<50%) expressed *Micu2* and *Mcub* suggesting that these markers were not very active in ECs or they are below the limit of detection (Figure 4c), and, hence, the significantly lower expression of *Mcub* in LCA versus RCA at 2D and 2W is of low importance (Figure 4c). Although it is not expected that mRNA levels will translate to equivalent protein levels, the *Mcu* upregulation by d‐flow compared to s‐flow supports an enhanced _m_Ca^2+^ uptake and establishment of the atheroprone EC phenotype under d‐flow. The functional significance of the *Mcur1* downregulation by d‐flow compared to s‐flow is currently undetermined.

## 4 DISCUSSION

**FIGURE 5:**
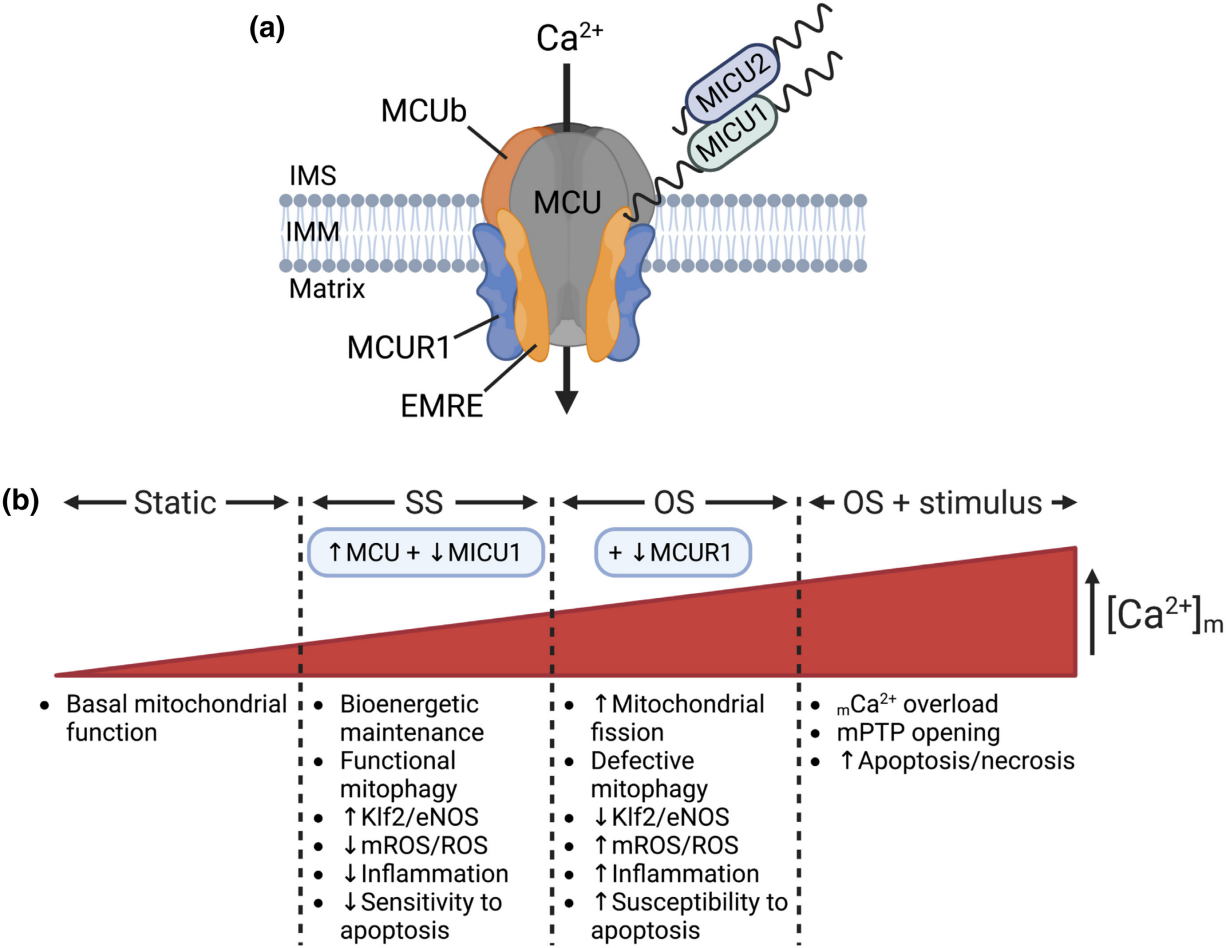
A schematic of the MCU complex with its subunits and a list of intracellular pathways by which the observed SS‐ and OS‐induced changes in MCU complex subunit expression levels may affect the complex activity and EC function. (a) The MCU channel is composed of four MCU subunits and up to four EMRE subunits, and is gated by the gatekeeper MICU1/2 dimers. MCUb can replace MCU subunits. MCUR1 forms a scaffolding part of the channel. (b) Based on the differential effects of each flow on the EC MCU complex subunit expression (this study) and on the EC redox state and key intracellular processes (literature; see text for additional information), it was proposed that the MCU complex activity changes as follows: Static < SS < OS < OS in the presence of an additional stimulus.

Our findings from cultured human ECs exposed to either pro‐atherogenic flow (OS) or anti‐atherogenic flow (SS) showed that both flows caused an upregulation of MCU and downregulation of the gatekeeper MICU1. Based on the structure/function of the MCU complex (Figure 5a), these changes would result in enhanced _m_Ca^2+^ uptake in ECs exposed to either flow compared to static controls (Figure 5b). A recent publication from our group verified that EC exposure to SS increases [Ca^2+^]_m_ levels compared to static ECs (Patel et al., 2022). In addition to a significant increase in MCU and a significant decrease in MICU1 expression relative to static, OS also caused a significant decrease in MCUR1 compared to SS or static. Due to conflicting reports on the MCUR1 function (Chaudhuri et al., 2016; Mallilankaraman et al., 2012a; Paupe et al., 2015; Tomar et al., 2016; Vais et al., 2015), it is difficult to assess the significance of this finding. However, the MCU complex activity/_m_Ca^2+^ uptake is expected to be higher in ECs under OS compared to SS based on the fact that (a) mROS (and cytosolic ROS levels) are higher under OS compared to SS (Dai et al., 2007; De Keulenaer et al., 1998; Hwang et al., 2003; Takabe et al., 2011) and (b) our group has shown that the MCU complex can sense an increase in mROS levels, via S‐glutathionylation of a conserved Cys97 in the MCU subunit, and increase its activity (Alevriadou et al., 2017, 2021; Dong et al., 2017).

Figure 5b summarizes what is currently known regarding the effects of SS and OS on mitochondrial and EC function: Prolonged SS is thought to maintain ΔΨ_m_ and the mitochondrial network morphology, and, via mitochondrial biogenesis (≥72 h), improve ATP production (Hong, Shin, Aldokhayyil, et al., 2022). OS in vitro and d‐flow in vivo resulted in increased mitochondrial fission compared to SS in vitro and s‐flow in vivo, respectively (Hong, Shin, Choi, et al., 2022), although not every report was in agreement (Coon et al., 2022). It is well accepted that SS results in active mitophagy, increases Klf2/eNOS, decreases mROS and ROS, suppresses inflammation and protects ECs from apoptosis in response to an additional stimulus, whereas OS causes the opposite trends (Coon et al., 2022; Dai et al., 2007; Hong, Shin, Aldokhayyil, et al., 2022; Scheitlin et al., 2014).

Importantly, regulation of MCU channel activity by the local hemodynamics was recently shown to play a critical role in EC mitophagy and inflammation (Coon et al., 2022): SS applied on cultured mouse aortic ECs was shown to activate Erk5, cause a transient increase followed by a sustained decrease in mitochondrial fragment counts (due to activation of mitophagy), upregulate *Klf2* and suppress *NF‐κB* transcriptional activity and inflammatory signaling. Deletion of the essential MCU complex gene *Emre* blocked the SS effects on EC signaling suggesting that the SS‐induced MCU‐mediated _m_Ca^2+^ influx, via activation of Erk5 kinase, mitophagy, and *Klf2*‐mediated gene expression (*eNOS* is a downstream transcriptional target of *Klf2*), regulates mitochondrial homeostasis and establishes the atheroprotective EC phenotype. These findings were verified in vivo by examining the endothelium under s‐flow (descending thoracic aorta) and d‐flow (inner aortic arch) of transgenic mouse models using en face immunofluorescence (Coon et al., 2022). It is already known that SS‐induced MCU‐mediated changes in [Ca^2+^]_m_ regulate the cytosolic Ca^2+^ signaling in ECs (Scheitlin et al., 2014) and other cells (Drago et al., 2012). Furthermore, [Ca^2+^]_m_/mROS are known to regulate mitochondrial dynamics (Giedt et al., 2012; Willems et al., 2015), mitochondrial dynamics determine mitophagy (Shirihai et al., 2015), and increased mitochondrial fission (interdependently with NF‐κB) induces EC inflammation (Forrester et al., 2020). A recent study showed increased mitochondrial fission in ECs exposed to d‐flow (inner aortic arch) compared to ECs under s‐flow (descending thoracic aorta) in a mouse model with EC‐specific photoactivatable mitochondria; these findings were verified in cultured ECs exposed to OS versus SS (Hong, Shin, Choi, et al., 2022). Based on Coon et al., it is now known that the SS‐regulated MCU activity is upstream of a specific kinase pathway, mitochondrial dynamics, mitophagy, Klf2 signaling, and EC inflammation. In their study, OS was found to induce fission equivalent to SS, but OS failed to activate mitophagy (less p62 upregulation and impaired assembly of the kinase‐ and p62‐dependent scaffolding complex required for mitophagy) leading to suppressed *Klf2* expression (Coon et al., 2022). An earlier study had reported excessive autophagy and an impaired autophagic flux under OS (Li et al., 2015). In either case, both the decrease in Klf2 signaling/NO production and the impaired clearance of fragmented mitochondria (a major source of ROS) will lead to the OS‐induced EC inflammation and dysfunction. None of the above findings has provided any information regarding the relative MCU complex activity in ECs under OS versus SS. However, ECs under OS showed [Ca^2+^]_c_ oscillations at a higher frequency than SS‐exposed ECs (Helmlinger et al., 1996), and [Ca^2+^]_m_ signaling was recently reported by us to be regulated by the MCU activity level in SS‐exposed ECs (Patel et al., 2022). Overall, based on the differential effects of OS versus SS on EC redox status, mitochondrial fission, mitophagy, and inflammation, and the known regulation of MCU complex activity by mROS, it is expected that OS will cause a significantly higher MCU activity than SS (Figure 5b); this may manifest as a higher oscillation frequency in the OS‐induced, compared to SS‐induced, EC [Ca^2+^]_m_ response.

Based on the recently summarized (Garbincius & Elrod, 2022) functions ascribed to MCUR1, it is conceivable that the OS‐induced decrease in MCUR1 expression (on top of the increase in MCU and decrease in MICU1 expressions that also occurred under SS) may play a causative role on the OS‐induced harmful effects on mitochondrial and EC function: If the loss in MCUR1 expression results in reduced ΔΨ_m_ (Paupe et al., 2015) and since a reduction in ΔΨ_m_ is critical for fission (Giedt et al., 2012), it would agree with the enhanced mitochondrial fission reported in OS‐ compared to SS‐exposed ECs (Hong, Shin, Choi, et al., 2022). Excessive fission might contribute to the defect in mitophagy and Klf2/eNOS signaling, and the resultant increase in oxidative stress would lead to NF‐κB‐mediated inflammation. The MCUR1 loss under OS, either independently (via its association with CypD) or as a result of the above changes, might also increase the EC susceptibility to mPTP opening and apoptosis. Additional research is needed on the role of MCUR1 in general, and its role in sheared ECs in particular.

There was a disagreement between expression at the mRNA and protein levels, as, for example, *MCU* and *MCUb* expression was found significantly higher in SS‐ compared to OS‐exposed ECs. Disagreement between mRNA and protein is attributed to mRNA degradation and posttranscriptional regulation of the MCU complex genes through miRs (Alevriadou et al., 2021; Garbincius & Elrod, 2022; Marchi & Pinton, 2014), and was previously reported in skeletal and cardiac muscle (Zaglia et al., 2017; Zampieri et al., 2016). Similarly, expression patterns of specific genes were different in vivo compared to in vitro: There was a significant upregulation of *Mcu* in mouse carotid ECs under prolonged d‐ versus s‐flow, which did not match the observed change in *MCU*. Both *MCUR1* and *Mcur1* were upregulated under SS and s‐flow, respectively, compared to OS and d‐flow. Disparities between in vivo and in vitro gene expression profiles may be due to the difference in species and vascular origin, that is, mouse carotid ECs versus HUVECs. However, a recent study found similar transcriptomic profiles between human aortic ECs and HUVECs when exposed to the same flows (Maurya et al., 2021). Furthermore, gene expression levels change with time; we measured at 24 h, as a representative time point for chronic flow application, and compared the in vitro data with in vivo data at 2D and 2W. Contrary to in vivo, in vitro models can expose cultured ECs to flows for a maximum of a few days. The difference between in vivo and in vitro gene expression may also be due to the complexity of the in vivo hemodynamic environment, as was described in recent reviews (Dessalles et al., 2021; Jackson et al., 2022). Two features of d‐flow that affect EC function are the presence of secondary/transverse flows, on top of the primary uniaxial flow, and spatial shear stress gradients that are missing from the in vitro OS. Besides luminal flow/shear stress, ECs in vivo experience transmural and interstitial flows/stresses, as well as tensile and normal stresses. They also reside on a softer substrate than a plastic or glass slide. The combination of all the above environmental factors will determine the intracellular signaling pathways, including those that regulate the MCU complex subunit expression and activity, and, ultimately, the EC (dys)function at a specific arterial location in vivo.

In summary, prolonged exposure to flow (24 h of SS or OS) led to an increase in MCU expression and a decrease in the gatekeeper MICU1 expression compared to static suggesting that either flow will cause increased MCU complex activity/_m_Ca^2+^ uptake. The most notable difference between SS and OS was the downregulation in MCUR1 expression under OS. The documented OS‐induced changes in mROS/ROS, mitochondrial fission, mitophagy, and susceptibility to apoptosis (especially, the increased mROS levels) support the notion of a differential increase in MCU complex activity/_m_Ca^2+^ uptake in OS‐exposed ECs compared to SS‐exposed ones. Based on the literature, the OS‐induced MCUR1 downregulation might play a role in regulating the sensitivity of mPTP to [Ca^2+^]_m_; it might either enhance the propensity to apoptosis or protect ECs from it. If we were to record [Ca^2+^]_m_ signals for a few minutes at the end of 24 h SS or OS, it would be difficult to attribute any differences in [Ca^2+^]_m_ signaling between SS‐ and OS‐conditioned ECs solely to the documented changes in MCU complex subunit expression, because prolonged SS and OS are known to differentially affect the expression and/or activities of additional players in flow‐regulated Ca^2+^ signaling. Specifically, SS, but not OS, redistributes Piezo1 from the cytosol to the basal membrane and activates the atheroprotective pathway of eNOS phosphorylation/NO production (Albarran‐Juarez et al., 2018; Lai et al., 2021), and also upregulates eNOS expression via Klf2 (Wang et al., 2010). Based on our recent work (Patel et al., 2022), [Ca^2+^]_m_ signaling in flow‐conditioned ECs will be the outcome of the combined SS‐ or OS‐induced changes in MCU complex, Piezo1, and eNOS activities (as well as any changes in the inositol trisphosphate receptor activity), and delineating the relative contribution of each of these effectors requires additional research. To demonstrate causality in linking the observed flow‐induced MCU complex subunit expression levels to EC phenotypes, future work should focus on genetically changing *MCU* or *MICU1* in combination with *MCUR1* and studying their effects on EC dysfunction prior to and following exposure to OS; in particular, whether MCUR1 overexpression can rescue the atheroprotective phenotype. Potential challenges are anticipated due to the fact that the OS and SS effects on gene/protein expression are subtle (neither complete ablation nor several‐fold overexpression), more than one subunit change their expression simultaneously, and any genetic changes may also affect the static phenotype and complicate the investigation into flow‐induced effects.

## AUTHOR CONTRIBUTIONS

A.P. designed experiments, acquired data, analyzed data, wrote and revised the manuscript. J.G.P. designed experiments, acquired data, analyzed data, wrote and revised the manuscript. M.V. acquired data, analyzed data. S.M. acquired data, analyzed data. J.E.B. analyzed data, wrote and revised the manuscript. M.M. conceived the project, revised the manuscript. B.R.A. conceived the project, designed experiments, analyzed data, wrote and revised the manuscript.

## FUNDING INFORMATION

No funding information provided.

## CONFLICT OF INTEREST

The authors have declared no conflict of interest.

## ETHICAL APPROVAL

The manuscript is a retrospective case report that does not require ethics committee approval at that institution. Basically, in this manuscript, we included our in vitro studies (using cultured cells) and an in silico analysis of a scRNA‐seq dataset that had been publicly archived by others (the dataset was from a mouse model of partial carotid ligation).
